# ROS-mediated thylakoid membrane remodeling and triacylglycerol biosynthesis under nitrogen starvation in the alga *Chlorella sorokiniana*

**DOI:** 10.3389/fpls.2024.1418049

**Published:** 2024-07-08

**Authors:** Jithesh Vijayan, Nishikant Wase, Kan Liu, Wyatt Morse, Chi Zhang, Wayne R. Riekhof

**Affiliations:** ^1^ School of Biological Sciences, University of Nebraska-Lincoln, Lincoln, NE, United States; ^2^ Department of Biochemistry, University of Nebraska-Lincoln, Lincoln, NE, United States; ^3^ Center for Plant Science Innovation, University of Nebraska-Lincoln, Lincoln, NE, United States; ^4^ PPD, part of ThermoFisher Scientific, Henrico, VA, United States; ^5^ Mayo Clinic, Rochester, MN, United States

**Keywords:** microalgae, oil accumulation, nitrogen limitation, ROS, lipid metabolism, membrane remodeling, biofuels

## Abstract

Many microbes accumulate energy storage molecules such as triglycerides (TAG) and starch during nutrient limitation. In eukaryotic green algae grown under nitrogen-limiting conditions, triglyceride accumulation is coupled with chlorosis and growth arrest. In this study, we show that reactive oxygen species (ROS) actively accumulate during nitrogen limitation in the microalga *Chlorella sorokiniana*. Accumulation of ROS is mediated by the downregulation of genes encoding ROS-quenching enzymes, such as superoxide dismutases, catalase, peroxiredoxin, and glutathione peroxidase-like, and by the upregulation of enzymes involved in generating ROS, such as NADPH oxidase, xanthine oxidase, and amine oxidases. The expression of genes involved in ascorbate and glutathione metabolism is also affected under this condition. ROS accumulation contributes to the degradation of monogalactosyl diacylglycerol (MGDG) and thylakoid membrane remodeling, leading to chlorosis. Quenching ROS under nitrogen limitation reduces the degradation of MGDG and the accumulation of TAG. This work shows that ROS accumulation, membrane remodeling, and TAG accumulation under nitrogen limitation are intricately linked in the microalga *C. sorokiniana.*

## Introduction

Microalgae are a potential source of renewable biofuels, as they are fast growing and do not compete with crops for arable land (Hu et al., 2008). Significant investments have been made in the past two decades toward developing algae as a reliable biofuel and bioproduct feedstock, by coupling algal biomass production to waste remediation (Allen et al., 2018). Microalgae, like many other microbes, accumulate storage molecules under unfavorable growth conditions (Zhu et al., 2016), and starch and oil are products of interest as sources of biofuel. Under nutrient limitation conditions, microalgae like *Chlorella* spp., Chlamydomonas (*Chlamydomonas reinhardtii*), and *Nannochloropsis* spp., accumulate oil (Miller et al., 2010; Stephenson et al., 2010; Wase et al., 2014; Ma X. N. et al., 2016; Jiang et al., 2019). Many efforts have been made to understand the mechanisms associated with metabolic remodeling and the regulation of oil accumulation during nitrogen (N) starvation (Miller et al., 2010; Wase et al., 2017; Wase et al., 2019). Many enzymes participating in the conversion of photosynthates into oil have been identified (Boyle et al., 2012; Shtaida et al., 2014). N limitation is a strong inducer of oil accumulation in different microalgae but often is associated with slower growth and severely compromised carbon fixation. This growth penalty constitutes an unfavorable factor when considering the industrial use of algae as a feedstock for waste remediation and biofuel production. Furthermore, growth arrest and accumulation of oil are accompanied by a decrease in chlorophyll content (Zhang et al., 2013) and chlorosis, also known as the “degreening” of cells.

Under N-limiting conditions, fatty acids for the biosynthesis of triacylglycerol (TAG) are derived from both a *de novo* synthesis route and by the remodeling of glycerolipids from preexisting membranes. Various enzymes involved in membrane remodeling have been reported in Chlamydomonas (Li et al., 2012a; Li et al., 2012b; Yoon et al., 2012) that act on different membrane glycerolipids to release fatty acid for TAG biosynthesis. One of the membrane lipids that experiences the most dramatic changes in abundance in response to N limitation is monogalactosyldiacylglycerol (MGDG), a plastid membrane lipid. Fatty acid from MGDG is channeled for triacylglycerol (TAG) synthesis (Li et al., 2012b).

All aerobic organisms use oxygen as an electron acceptor for respiration. Various pathways lead to the production of reactive oxygen species (ROS), such as mitochondrial electron transport, nitric oxide synthase, and peroxisomal β-oxidation (Møller, 2001). Different forms of ROS accumulate in cells, such as superoxide (O_2_^–^), hydroxyl radical (•OH^–^), singlet oxygen (^1^O_2_), and hydrogen peroxide (H_2_O_2_). Each of these reactive species have different half-lives and chemical properties that affect cellular biochemistry and physiology (Foyer and Noctor, 2009).

Cellular redox and ROS homeostasis are carefully balanced by an elaborate antioxidant defense system that includes the enzymes superoxide dismutase (SOD), catalase, glutathione peroxidase, and ascorbate peroxidase as well as low-molecular-weight scavengers such as uric acid, ascorbate, glutathione, carotenoids, and flavonoids (Czarnocka and Karpiński, 2018). Studies over the past two decades in plants and animals have revealed ROS as a fine-tuned signaling system that elicits both localized and global responses. Under different biotic and abiotic stress conditions, ROS signaling has been implicated in playing a key role in stress responses and acclimation (Ben Rejeb et al., 2015; Gupta et al., 2013; He et al., 2017) in both animals and plants (Foyer and Noctor, 2009; Schieber and Chandel, 2014).

In this study, we provide evidence that ROS signaling participates in the N starvation response in *Chlorella sorokiniana*, an industrial microalga of the *Chlorophyceae* family. We show that *C. sorokiniana* uses transcriptional mechanisms to control ROS accumulation and that ROS signaling is essential for chloroplast membrane remodeling, leading to chlorosis and oil accumulation.

## Materials and methods

### Cultures and growth measurements

*Chlorella sorokiniana* cells were grown in Tris acetate phosphate (TAP) medium (Gorman and Levine, 1965) at 25°C under continuous light of 40 μE. For nitrogen (N) limitation, parental cultures were grown for 2 days and then washed three times in the appropriate medium (TAP or TAP-N) before inoculation at a final concentration of 10^7^ cells/mL. Cells were inoculated into either control (TAP) or N-limited medium of 10 mM or 250 µM ammonium chloride, respectively. Culture growth was recorded as optical density (OD) at 750 nm.

### Nile red staining for oil droplets

*C. sorokiniana* cells grown in appropriate conditions were first adjusted to an OD of 0.2 in respective media before 10 µL of a 100 µg/mL stock of Nile red (Sigma Aldrich) in DMSO was added to 100 µL of diluted cell suspension. After 10 min of incubation, the cells were visualized by fluorescence microscopy and fluorescence intensity was measured. Microscopy was conducted using an EVOS-fl epifluorescence microscope with a GFP light cube. Quantitative fluorescence measurements were performed on a Synergy H1 hybrid reader with excitation and emission wavelengths of 455 nm and 560 nm, respectively; fluorescence values were normalized to the optical density.

### Transmission electron microscopy analysis

For transmission electron microscopy (TEM) analysis, cells were grown in the appropriate medium for 4 days and then fixed in 1% formaldehyde before processing the samples. Sample preparation and analysis were carried out as described by Gojkovic et al. (2014.) Imaging was carried out on a Hitachi H-7500 microscope.

### Radiochemical labeling and analysis of lipids

Two days after inoculation, parental cultures were supplemented with ^14^C acetate (10^6^ cpm/mL of culture-specific activity of 56 mCi/mmol, supplied by ARC Inc.) for 8 h. The cells were washed and inoculated in appropriate medium (TAP or TAP-N) at a concentration of 10^7^ cells/mL. One milliliter of culture was collected at each time point, including at time zero. Cell pellets were collected by centrifugation at 4,000 rpm for 5 min at 25°C. Bligh and Dyer extraction was performed on the samples by partitioning lipids into the organic phase of a chloroform/methanol/0.2 M KCl solvent system (1:1:0.8, v/v/v). Glycerolipids were dried under N_2_ and sequentially separated by thin-layer chromatography (TLC) on a silica G60 plate (EMD-Millipore) in two solvent systems. The first ascent was performed until two-thirds the height of the plate in a solvent system of chloroform, methanol, acetic acid, and water (85:12.5:12.5:3, v/v/v/v) to separate polar lipids. The plate was then dried, and a second ascent to the full height of the plate was performed in petroleum ether, diethyl ether, and acetic acid (80:20:1) to separate neutral lipids such as TAG, steryl esters, and free sterols. After drying, plates were exposed to a storage phosphor screen for 24 h, and radioactivity was detected on a GE-Typhoon FLA 9500 scanner. Images were analyzed by ImageQuant TL v8.1, and individual bands were identified by reference to standards of known R_f_ and plotted as a percentage of initial counts in the specific lipid.

### Transcriptome deep sequencing (RNA-seq) analysis

For RNA-seq analysis, two biological replicates grown at the same time but from different parent cultures in different flasks (independently grown cultures) were used. Samples were collected by pelleting the cells at 5,000 rpm for 3 min before freezing them in liquid nitrogen. Total RNA was extracted with the TRIzol reagent according to the manufacturer’s protocol. Briefly, frozen cells were gently resuspended in 500 µL of TRIzol reagent (Ambion TRIzol LS reagent), to which 600 µL of chloroform was added, gently mixed, and phase-separated at 12,000 rpm for 10 min at 4°C. Total RNA was precipitated by adding 40 µL of 5 M NaCl and 500 µL isopropanol to the supernatant and incubating at 4°C for 10 min. RNA was pelleted by centrifugation at 13.8 × g for 20 min at 4°C, followed by washing the pellet with ethanol and further centrifugation for 3 min at 13.8 × g in 4°C. The pellet was carefully dried and resuspended in RNase-free water. RNA sequencing library preparation and Illumina sequencing (75-bp, single-end reads) were carried out by GeneWiz (https://www.genewiz.com/). Two separate libraries were prepared and sequenced on different lanes per sample. Libraries were prepared using the Illumina TruSeq RNA sample preparation kit according to the manufacturer’s guidelines. Reads were aligned to our in-house genome sequence of *C. sorokiniana* UTEX 1230 (tentative id: SUB1977110) using the Rsubread package (Liao et al., 2013). Reads were mapped and counted using the featureCounts function with default setting. Differential expression analysis was performed using the edgeR-limma package. Weighted trimmed mean of M-values (TMM) were used to normalize the count data. Firstly, low-expressing transcripts were filtered out and 15,382 transcripts were retained. The linear model was fitted and differential expression analysis was performed using the eBayes function of limma package, and expression data for all genes are included as **Supplementary Data File 1** (Robinson et al., 2009; Ritchie et al., 2015). Raw reads have been submitted to NCBI under the BioProject with accession number PRJNA1111962.

### ROS assay

The quantification of ROS was carried out using 2′, 7′-dichlorodihydrofluorescein diacetate (DCFDA) cellular ROS detection assay kit (Abcam, product number AB113851) following the manufacturer’s protocol. Briefly, cells under different treatments were washed twice in the assay buffer and then adjusted to an OD of 0.2 before incubation with DCFDA at a final concentration of 10 µM for 1 h. The cells were then washed twice with the appropriate medium (TAP or TAP-N) to remove excess dye and incubated for one more hour. Fluorescence intensity was measured with excitation and emission wavelengths of 485 nm and 535 nm, respectively on a BioTek SynergyH1 reader. Values were normalized to the optical density.

### Inhibitors and supplements

A working solution of 250 µM of diethyldithiocarbamate (Sigma-Aldrich) solubilized in DMSO was used to inhibit superoxide dismutase and induce ROS accumulation. N-limited medium was supplemented with 500 µM ascorbate (Sigma Aldrich) to quench hydrogen peroxide. NADPH oxidase was inhibited by the addition of 50 µM 2-acetylphenothiazine (ML171, Selleck Chem) dissolved in DMSO.

## Results

### Nitrogen limitation causes growth arrest coupled with chloroplast membrane remodeling and oil accumulation

**Figure 1:**
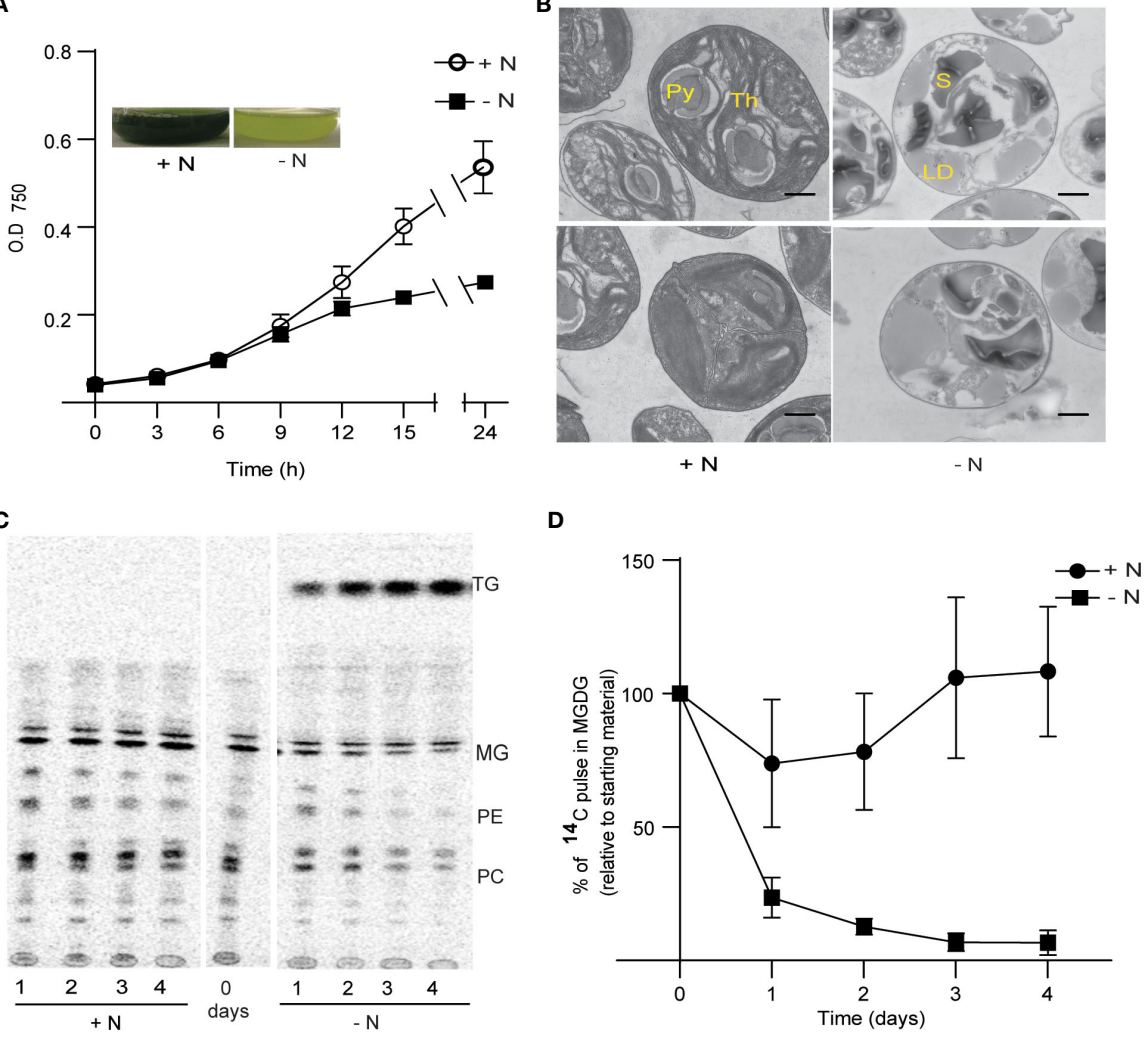
Growth and thylakoid membrane remodeling in *Chlorella sorokiniana* under nitrogen limitation. **(A)** Growth curves of *Chlorella sorokiniana* in N-replete (N+, 7.5 mM) and N-limited media (N−, 0.25 mM). Cultures were inoculated with 10^7^ cells/ml, and the mean +/− SD of three biological replicates is presented. Inset: representative cultures under N-replete (N+) and N-deplete (N−) condition after 48 h of growth. **(B)** TEM image of *Chlorella sorokiniana* cells under N+ or N− conditions 4 days post inoculation. Scale bar indicates 800 nm. **(C)** Representative TLC radiograph of cells prelabeled with ^14^C acetate and then grown in N+ or N− media, indicating the degradation of preformed lipids under N− conditions. TAG, triacylglycerol; MG, monogalactosyldiacylglycerol; PE, phosphatidylethanolamine; PC, phosphatidylcholine. Lipid droplet accumulation under N limitation. **(D)** Quantitative measurement of ^14^C-containing MGDG degradation upon N limitation. Measurements are relative to the amount to ^14^C pulse present in MGDG at the initial time point (t0).

Nitrogen (N) is an essential macronutrient for the growth of all organisms, as it provides the building blocks for the biosynthesis of nucleic acids, amino acids, and the amino acid–derived head groups of certain lipids like phosphatidylcholine and phosphatidylethanolamine. N limitation led to growth arrest of *Chlorella sorokiniana* (**Figure 1A**). We observed a decrease in growth as early as 9 h after inoculation into N-limited (250 µM ammonium chloride) acetate-containing medium, compared with cells grown in N-replete medium (10 mM ammonium chloride). *C*. *sorokiniana*, like many other microalgae, accumulated oil droplets when deprived of N, as illustrated by the green puncta observed specifically in N-limited *C. sorokiniana* cells stained with Nile red (**Supplementary Figure 1A**). N limitation was also associated with chlorosis in *C. sorokiniana* (**Figure 1A**, inset).

Chlorosis is generally caused by the degeneration of chloroplast membranes and is accompanied by chlorophyll degradation (Li et al., 2012b). We thus carried out transmission electron microscopy (TEM) observations of the ultrastructure of chloroplast membranes (**Figure 1B**). We observed a decrease in the number of dense thylakoid membranes in N-starved cells (after 4 days), relative to the control cells (N+). One of the characteristics of N limitation in Chlamydomonas is the substantial reduction in MGDG levels (Li et al., 2012b). To assess the extent of MGDG degradation under N limitation in *C. sorokiniana*, we labeled membrane lipids from N-replete cultures to saturation with 1,2-^14^C-acetate. We determined that MGDG is degraded when cells are transferred to N-limited conditions, as seen during a chase period with the replacement of labeled acetate with unlabeled acetate (**Figures 1C, D**). Other lipids, such as phosphatidylcholine and phosphatidylethanolamine, also appeared to be degraded upon transfer to N limitation, but not when cells were maintained in N-replete medium. Fatty acids released from these lipids were channeled into TAG, as evidenced by the increase in radiolabeled TAG during the N-limited chase period (**Figure 1C**).

### A transcriptomic signature of ROS accumulation under N limitation

To explore the mechanisms underlying chloroplast membrane degradation and oil accumulation under N limitation, we performed a transcriptome deep sequencing (RNA-seq) analysis. Accordingly, we collected cells grown in N-replete conditions or transferred to N-limited conditions for 9 h, when we first observed a decline in growth rate relative to N-replete cultures (**Figure 1A**); we also collected cells after 24 h in N-limited conditions as a late time point. We determined that 16% of all genes are differentially expressed 9 h into N limitation and reached 22% at the 24-h time point (**Table 1**). Differentially expressed gene sets of 9 h and 24 h significantly overlap (**Supplementary Figure 2**). The substantial rewiring of the transcriptome observed here indicated that the N starvation response in *C. sorokiniana* starts earlier than 9 h after the onset of N limitation, i.e., before a significant reduction in growth is noticeable. This is expected as nutrient sensing and initiation of starvation response would precede the decrease in growth rate.

**Table 1:** Summary of differentially expressed genes.

Condition	N vs. T-9h (%)	N vs. T-24h (%)
Down	1,076 (6.99%)	1,693 (11%)
No significant change	12,805	11,895
Up	1,501 (9.76%)	1,794 (11.66%)
Total	15,382	15,382

Number of genes whose expression significantly changed between the indicated growth conditions, as determined by an adjusted P-value of <0.05 and Log_2_(fold-change) of ≥2. The numbers in parentheses indicate the percentage relative to the total number of genes.

**Figure 2:**
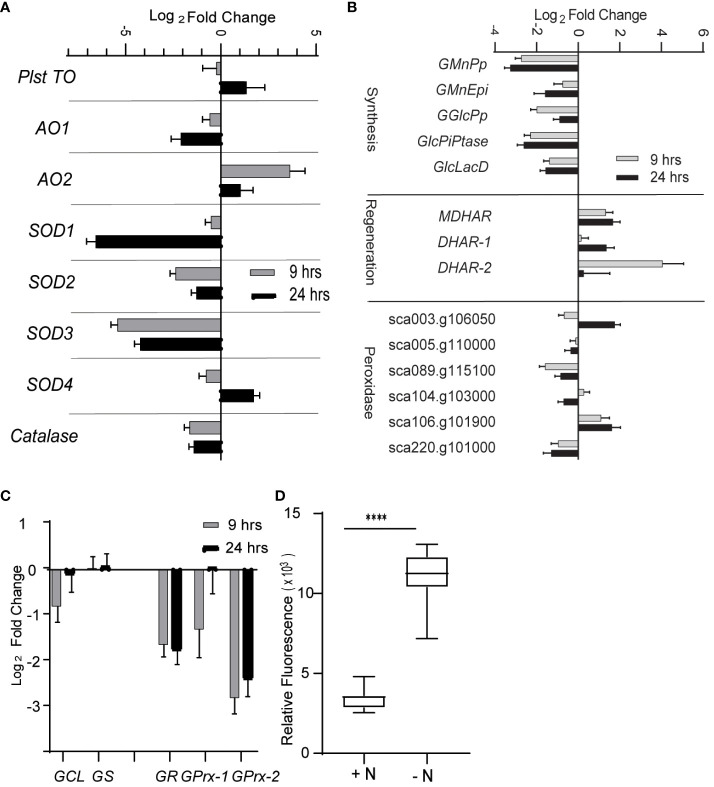
Reactive oxygen species under nitrogen limitation. **(A)** Expression of enzymes involved scavenging ROS. AO, alternative oxidase; SOD, superoxide dismutase; Plst TO, plastidial terminal oxidase. **(B)** Expression of genes involved in ascorbate (AsA) metabolism. Genes are classified as those involved in AsA synthesis, regeneration, and AsA-dependent peroxidases. GMnPp, GDP-d-mannose pyrophosphorylase; GMnEpi, GDP-mannose 3,5-pimerase 1; GGlcPp, GDP-L-galactose phosphorylase 1; GlcPiPtase: L-galactose-1-phosphate phosphatase; GlcLacD, L-galactono-1,4-lactone dehydrogenase; MDHAR, monodehydroascorbate reductase; DHAR, dehydroascorbate reductase. **(C)** Expression of genes involved in glutathione metabolism. GSH1, gamma-glutamylcysteine synthetase; GSH2, glutathione synthetase are involved in glutathione synthesis; GR, glutathione reductase regenerates reduced glutathione (GSH) from oxidized form of glutathione (GSSG); GPrx, glutathione peroxidases oxidizes GSH to quench ROS and form GSSG. **(D)** ROS level as measured by DCFDA fluorescence under N-limited condition after 24 h of inoculation in appropriate media. The values are normalized to optical density. Error bars indicate SD (n = 3). **** P value < 0.0001.

From the RNA-seq data, we determined that the expression of many genes encoding enzymes involved in quenching ROS are decreased during N limitation. Indeed, the expression of superoxide dismutase genes (*SOD1*, *SOD2*, and *SOD3*) and catalase genes was decreased at 9 h and 24 h into N-limited conditions (**Figure 2A**). Enzymes such as SOD, catalase, and glutathione peroxidase quench and detoxify ROS generated during various cellular processes such as photosynthesis and mitochondrial electron transport (Møller, 2001; Asada, 2006). SOD converts superoxide radicals to H_2_O_2_, which is further degraded into less harmful substances by a variety of enzymes, such as peroxidases and catalases (Czarnocka and Karpiński, 2018).

The expression of genes involved in the metabolism of ascorbate (AsA), a key antioxidant, was also significantly altered (**Figure 2B**). Specifically, genes involved in AsA biosynthesis from GDP-mannose were decreased in abundance under N limitation, including those encoding GDP-d-mannose pyrophosphorylase (GMnPp), GDP-mannose-3,5-epimerase 1 (GMnEpi), GDP-L-galactose phosphorylase 1 (GGlcPp), L-galactose-1-phosphate phosphatase (GlcPiPtase), and L-galactono-1,4-lactone dehydrogenase (GlcLacD) (Smirnoff, 2018). This observation suggested that AsA biosynthesis possibly diminishes upon N limitation. Conversely, the expression of genes encoding AsA-reducing enzymes such as monodehydroascorbate reductase (MDHAR) and dehydroascorbate reductase (DHAR) was upregulated in N-limited conditions (**Figure 2B**), indicating that the lower availability of AsA may be partially compensated for by increased regeneration of oxidized monodehydroascorbate or dehydroascorbate. *Glutathione reductase* (*GR*) expression, whose encoding enzyme regenerates glutathione (GSH) by reducing its oxidized form GSSG using NADH as a substrate (Foyer and Noctor, 2009), was also decreased in N-limited cells (**Figure 2C**). The expression of *glutathione peroxidase-like* genes (*GPrxL-1* and *GPrxL-2*) was lower in N-limited cells compared with N-replete cells; the encoded enzymes possibly use GSH to reduce H_2_O_2_. This result indicated that GSH-mediated quenching of H_2_O_2_ may be lower in *C. sorokiniana* N-limited cells as well.

Decreased transcript abundance of genes encoding key enzymes involved in ROS quenching suggested that *C. sorokiniana* cells experiencing N limitation accumulate ROS in a deliberate, transcriptionally regulated manner. To test this hypothesis, we measured intracellular ROS levels in N-limited cells using the cell-permeable ROS-sensitive fluorescent dye 2′-7′-dichlorodihydrofluorescein diacetate (DCFDA). Indeed, we determined that ROS levels are significantly higher in N-limited *C. sorokiniana* cells than in the control cells (**Figure 2D**).

To gain a more complete picture of redox regulation under N limitation, we analyzed the expression profile of various genes encoding members of the ferredoxin, peroxiredoxin, thioredoxin, and glutaredoxin families. We observed significant changes in the expression of many *Ferredoxin* genes (**Table 2**). Their encoded proteins can function as a reservoir of redox potential, accepting or donating electrons to various biochemical processes, including photosynthesis (Schürmann and Buchanan, 2008). The expression of four *Peroxiredoxin* genes was significantly decreased upon N limitation (**Table 2**). Along with catalases and peroxidases, peroxiredoxins scavenge hydrogen peroxide (Rhee and Woo, 2011). The lower expression levels of *Peroxiredoxin* genes suggested that yet another mechanism of ROS scavenging might be perturbed in cells exposed to N limitation. The expression of many *Thioredoxin* genes also changed upon N limitation (**Table 2**). Thioredoxins contribute to cell signaling by modifying cysteine residues in proteins (Baumann and Juttner, 2002). We also noticed decreased abundance of genes encoding thioredoxin-reducing enzymes such as ferredoxin-thioredoxin reductases 1 and 2 (Frdxn TrxRed1 and 2) (Schürmann and Buchanan, 2008) (**Table 2**), indicating that thioredoxin-mediated redox signaling is altered under N-limitation conditions. Genes encoding glutaredoxins, which share functional similarity to thioredoxins (Meyer et al., 2008), were also differentially regulated (**Table 2**). However, we identified these genes based on sequence similarity to previously characterized members of this gene family; determining their putative function requires further characterization and validation.

**Table 2:** Expression of genes encoding redox proteins during nitrogen limitation.

	Locus	N9		N24			Locus	N9		N24	
		LFC	SD	LFC	SD			LFC	SD	LFC	SD
FERREDOXIN	sca016.g102050	**–4.88**	0.31	**–7.73**	0.47	THIOREDOXIN	sca099.g100150	**–4.95**	0.55	**–7.8**	1.02
	sca167.g100250	**–3.57**	0.33	**–3.78**	0.35		sca110.g105350	**–1.9**	0.49	**–3.08**	0.73
	sca084.g106200	**–3**	0.34	**–2.92**	0.32		sca031.g101200	**–3.24**	0.37	**–2.62**	0.39
	sca084.g104400	**–2.63**	0.37	**–1.8**	0.39		sca022.g103350	**–2.62**	0.27	**–2.52**	0.28
	sca005.g104500	**–2.72**	0.27	**–1.71**	0.26		sca011.g104800	**–2.2**	0.26	**–2.08**	0.26
	sca214.g100950	**–1.73**	0.36	**–1.54**	0.41		sca033.g106700	**–2.5**	0.27	**–1.78**	0.26
	sca181.g103050	**–1.62**	0.27	**–1.2**	0.26		sca035.g103300	**3.53**	1.08	–1.26	1.40
	sca270.g100350	**–1.51**	0.26	**–1.16**	0.25		sca163.g106250	**–1.62**	0.26	**–0.87**	0.26
	sca084.g103950	–0.19	0.45	0.25	0.44		sca193.g103850	**–1.01**	0.30	–0.41	0.30
	sca035.g101100	–1.14	0.25	0.55	0.26		sca106.g107100	0.17	0.43	–0.23	0.52
	sca013.g100600	1.15	0.28	1.26	0.29		sca044.g100650	3.1	0.88	0.1	1.27
	sca070.g100250	**2.54**	0.40	**1.52**	0.36		sca104.g108850	–0.03	0.45	0.13	0.44
	sca225.g100750	**2.05**	0.29	**2.44**	0.29		sca217.g101750	–0.57	0.33	0.24	0.37
	sca089.g117450	**4.7**	0.56	**6.74**	0.58		sca110.g107100	2.81	1.32	0.42	1.68
PEROXIREDOXIN	sca163.g100950	**–6.98**	0.57	**-8.95**	0.95		sca267.g101550	0.03	0.25	0.48	0.26
	sca179.g100450	**–2.9**	0.27	**-2.75**	0.27		sca126.g101050	**1.55**	0.47	**0.84**	0.45
	sca044.g101300	**–2.48**	0.27	**-2.69**	0.27		sca139.g113050	0.45	0.27	**1.55**	0.28
	sca174.g100700	**–2.73**	0.47	**-2.1**	0.49		sca084.g103750	–0.52	0.35	**1.72**	0.34
	sca114.g103950	0.78	1.11	-0.85	1.60		sca081.g100250	1.5	0.57	2.1	0.73
	sca123.g101250	2.14	1.28	-0.75	1.56		sca193.g103550	**0.9**	0.26	**2.14**	0.27
	sca106.g102950	**–0.97**	0.32	-0.36	0.35		sca001.g104150	**2.38**	0.30	**2.39**	0.29
	sca163.g100900	**0.6**	0.26	**0.96**	0.27		sca163.g105750	**3.02**	0.56	**3.7**	0.58
GLUTAREDOXIN	sca134.g101400	**–1.75**	0.29	**–1.48**	0.30		sca049.g101050	**3.19**	0.41	**4.95**	0.43
	sca211.g100200	**–1.32**	0.25	**–1.05**	0.25		*Frdxn TrxRed 2*	**–3**	0.28	**–2.33**	0.26
	sca035.g102800	**–1.26**	0.27	**–0.9**	0.26		*Frdxn TrxRed 1*	**–3.17**	0.28	**–2.13**	0.26
	sca017.g103100	**–1.05**	0.36	**–0.42**	0.35		*Trx Prx*	0.34	0.29	**–1.46**	0.39
	sca217.g101850	**–0.75**	0.29	**–0.32**	0.29		*Frdxn-NADPH*	**–0.95**	0.25	**–1.23**	0.25
	sca238.g101050	–0.16	1.33	1.15	1.21						
	sca050.g100650	**2.53**	0.83	1.36	0.93						
	sca056.g101750	0.1	0.26	**1.49**	0.27						
	sca035.g108250	**1.29**	0.27	**2.18**	0.29						

Log fold-changes shown in bold are statistically significant (P ≤ 0.05). LFC, Log_2_(fold-change); SD, standard deviation; Frdxn TrxRed, ferredoxin-dependent thioredoxin reductase; Trx Prx, thioredoxin peroxidase; Frdxn-NADPH, NADPH-dependent thioredoxin reductase.

In light of the observation that genes encoding SODs, catalase, peroxidases, ferredoxins, and peroxiredoxins are downregulated upon N limitation, we propose that ROS accumulation in N-limited *C. sorokiniana* cells is mediated, at least partially, by the downregulation of ROS scavenging enzymes leading to a more oxidized cellular environment.

### ROS and chloroplast remodeling

**Figure 3:**
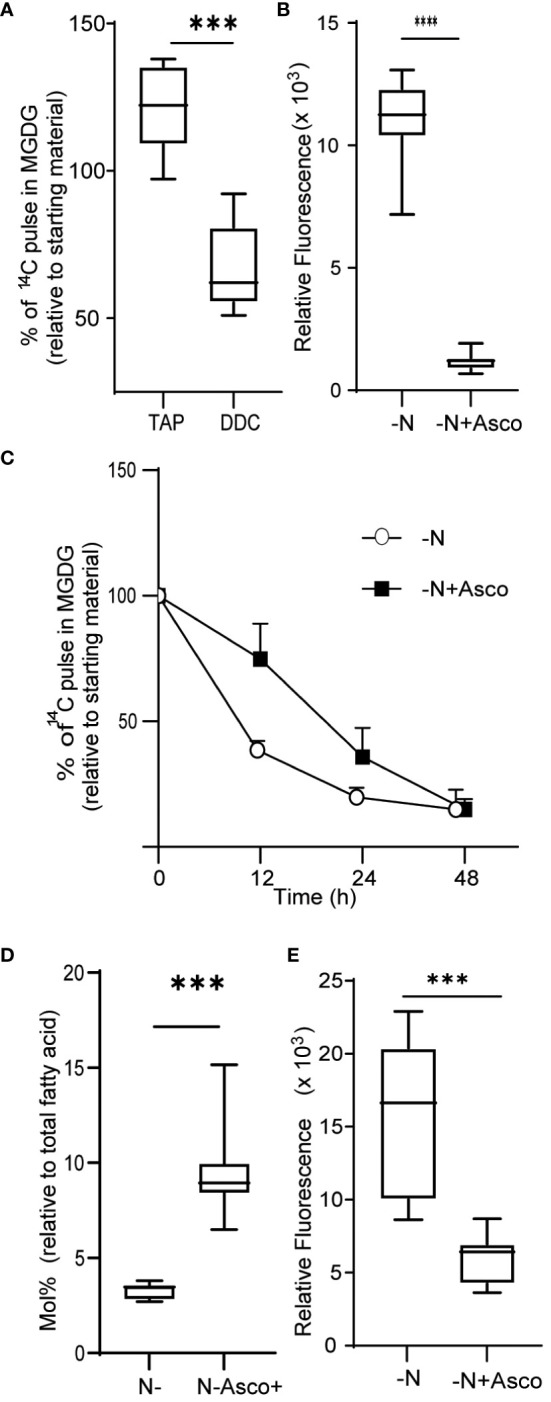
Quenching of ROS under nitrogen limitation and induction of ROS under nitrogen replete conditions. **(A)** Quantitative measurement of MGDG degradation in nitrogen replete media (TAP) when treated with 250 µM diethyldithiocarbamate (DDC) for 24 h. **(B)** ROS level under nitrogen-limited media fed with ascorbate, as measured by DCFDA fluorescence after 24 h of inoculation. Fluorescence values are normalized to O.D. **(C)** Quantitative measurement of MGDG degradation under N lim media when fed with 500 μM ascorbate. **(D)** Mole percentage of MGDG relative to total fatty acid from cultures under N limitation with or without ascorbate. **(E)** Level of TAG accumulation as measured by Nile red, under nitrogen limitation after 24 h of inoculation when fed with ascorbate. In all experiments n>7. Asterix indicates the statistical significance of the student t-test. *** indicates <0.001 and **** indicates <0.0001.

We next asked if the observed increase in ROS levels contributed to the degradation of MGDG and other membrane remodeling events observed during N-limiting growth. To this end, we treated cells grown under N-replete conditions with diethyldithiocarbamate (DDC), a known inhibitor of SOD (Heikkila et al., 1976). Treatment with DDC induced ROS accumulation (**Supplementary Figure 3**). Treatment of cells with 250 µM DDC resulted in MGDG degradation (**Figure 3A**), although not to the same extent as in N-limited cells. The conversion of superoxide to hydrogen peroxide catalyzed by SOD can also occur spontaneously at a slower rate, and other ROS quenching mechanisms are active as well, which may decrease the accumulation of ROS relative to that of N-limited cells. Conversely, we wished to test if preventing the accumulation of ROS under N-limitation conditions might prevent membrane remodeling. To test this hypothesis, we supplemented the medium with 500 µM ascorbate to quench cellular hydrogen peroxide. We established that ROS levels are lower in ascorbate-treated cells 24 h after the onset of N-limitation (**Figure 3B**). These lower ROS levels under N limitation were coupled with a decrease in MGDG degradation (**Figure 3C**). A time-course analysis indicated that while N-limited cultures supplemented with ascorbate still degraded MGDG, their rate of degradation was slower than that of control N-limited cultures (**Figure 3C**). This is congruent with the observation that MGDG levels are higher in N-limited cultures when treated with ascorbate (**Figure 3D**). We also noticed that TAG accumulates to lower levels in N-limited cells treated with ascorbate after 24 h of N limitation (**Figure 3E**). Taken together, these results indicate that ROS accumulation under N limitation plays a vital role in chloroplast membrane remodeling degradation of MGDG and channeling fatty acids for TAG biosynthesis.

### Sources of ROS under N limitation

**Figure 4:**
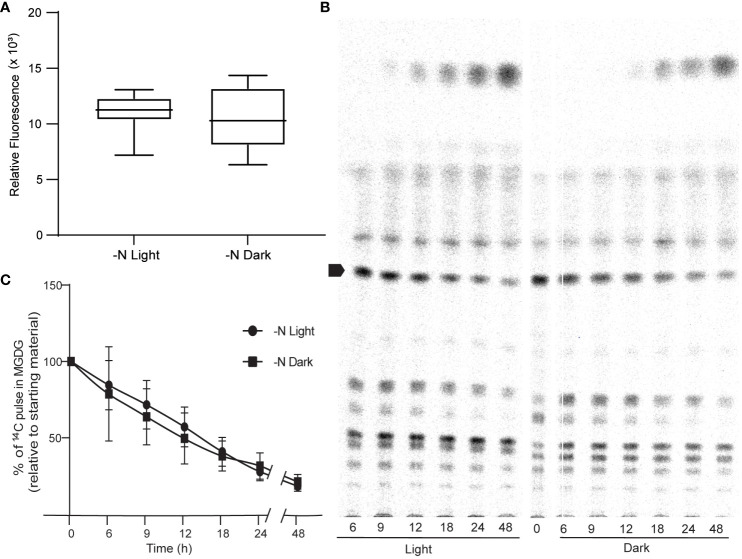
Photosynthesis as a source of ROS. **(A)** ROS levels of N lim culture grown under light (N-lim-L) and dark (N-lim-D) regimes as measured by DCFDA fluorescence after 24 h of inoculation. Fluorescence relative to O.D. **(B)** A representative TLC radiograph showing the remodeling of different lipids under nitrogen limitation in light or dark regime. Arrow indicates MGDG. **(C)** Quantitative measurement of degradation of preformed MGDG relative to the starting levels of 14C pulse in MGDG in N-lim cultures grown in light and dark regime. Error bars indicate SD (n = 3).

We sought to identify the possible source of ROS. Photosynthesis is a prominent source of superoxide in all photosynthetic organisms (Czarnocka and Karpiński, 2018). We thus tested the hypothesis that the increase in ROS levels observed in N-limited cells is derived from photosynthesis. To this end, we measured ROS levels in cells grown under continuous light or continuous darkness in N-limited conditions. We predicted that ROS levels would be lower in N-limited cells grown in the dark. However, we measured no appreciable difference in ROS levels between light- and dark-grown cells (**Figure 4A**). This result was also reflected in the very similar lipid profiles of the two types of cultures, as revealed by autoradiography after thin-layer chromatography separation of lipids (**Figure 4B**). We also quantified 1,2-^14^C-acetate-labeled MGDG levels from the autoradiograms and observed no differences between light- and dark-grown cultures (**Figure 4C**). These results collectively indicated that ROS involved in lipid remodeling in *C. sorokiniana* are from light-independent sources.

**Figure 5:**
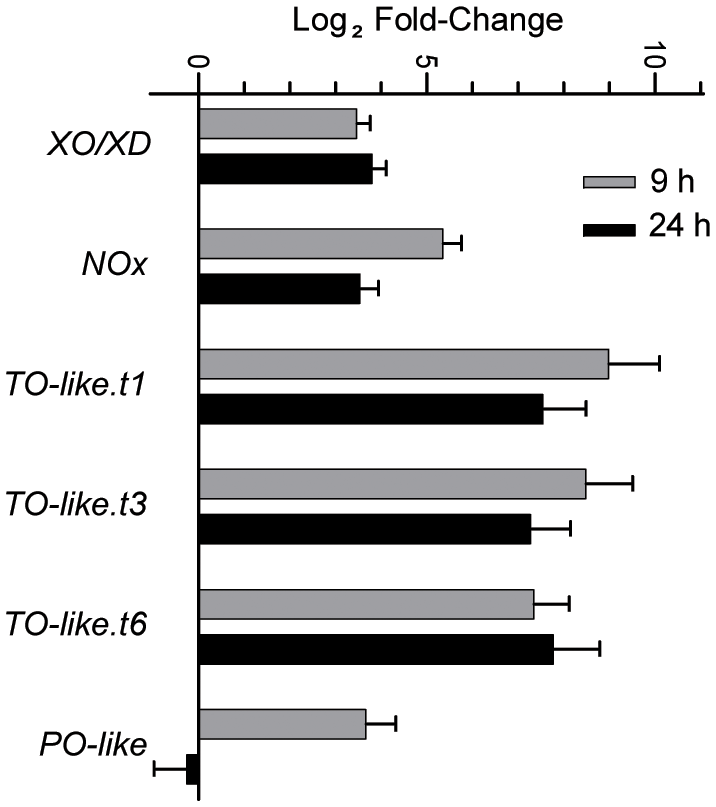
Expression of different ROS generating enzymes that increase during N-limited growth as detected in the RNA-seq studies. Error bars indicate SD (n = 2). XO/XD, xanthine oxidase/dehydrogenase; NOx, NADPH oxidase; TO-like, tyramine oxidase-like; PO-like, putrescine oxidase-like. TO-like.t1, t3 and t6 indicate the three different splice variants encoded by the same loci.

We next queried the transcriptome to identify genes encoding ROS-generating enzymes that are differentially expressed under N limitation. We determined that the expression of genes such as *NADPH oxidase*, *Xanthine oxidase/dehydrogenase*, *Putrescine oxidase-like*, and *Copper amine oxidase* (*Tyramine oxidase-like*) is upregulated under N limitation (**Figure 5**). The encoded enzymes all catalyze reactions that produce hydrogen peroxide or superoxide (Ma X. et al., 2016; Tavladoraki et al., 2016; Zandalinas and Mittler, 2018). Thus, the altered gene expression profiles we observed in genes with assigned roles in ROS generation suggested that ROS accumulation is enhanced by both the repression of ROS-quenching activities and the induction of ROS-generating activities.

### The role of NADPH oxidase in membrane remodeling

**Figure 6:**
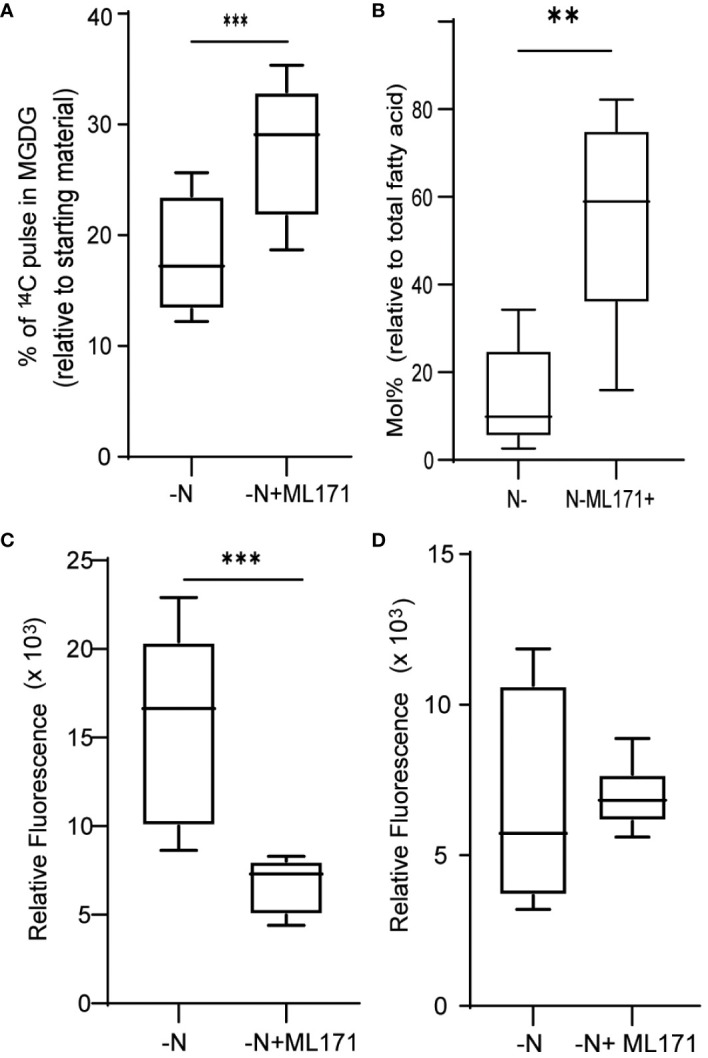
Role of NADPH oxidase in ROS generation and membrane remodeling. **(A)** MGDG degradation as measured by the remaining amount of preformed MGDG (24 h after inoculation), under nitrogen limitation when treated with 50 µM ML171-NADPH oxidase inhibitor. **(B)** Mol% of MGDG relative to total fatty acids in the cultures under N limitation treated with or without ML171. **(C)** TAG levels, measured as Nile red fluorescence, at 24 h post inoculation in cultures treated with ML171. **(D)** ROS levels, as measured by DCFDA fluorescence, under N-lim condition when treated with, ML171 (50 µM). ROS assay was carried out after 24 h of inoculation. Fluorescence is normalized to O.D. All error bars indicate S.D (n = 3). ** indicates a p-value < 0.001 and *** Indicates a p-value < 0.0001 in a paired student t-test.

NADPH oxidases (NOXs) are well-studied enzymes due to their various physiological roles in human health. Their functions are well researched in mammalian systems and are important drug targets to counteract various disorders (Meitzler et al., 2014). Multiple NOX inhibitors are therefore available to manipulate NOX activity levels (Altenhöfer et al., 2015). Here, we used 2-acetyl phenothiazine, also known as ML171, to inhibit NOX activity and, thus, NOX-mediated ROS production (Gianni et al., 2010). When cultures were treated with 50 µM ML171 under N limitation, we observed a decrease in MGDG degradation (**Figure 6A**). Decrease in MGDG degradation leads to increased amount of MGDG as a percentage of total lipids (**Figure 6B**). This is coupled with lower TAG accumulation (**Figure 6C**). However, we did not see a significant decrease in ROS levels (**Figure 6D**), as measured by DCFDA fluorescence. The fact that MGDG degradation and TAG accumulation are decreased upon treatment with ML171 indicated that NADPH oxidase may play a role in the N starvation response.

## Discussion

Macronutrient limitation has been studied for decades as a potent inducer of storage compound (oil and starch) accumulation in oleaginous photosynthetic microorganisms. However, the industrial application of this mechanism of oil accumulation is invariably limited by lower photosynthetic capacity and biomass yield. Finding ways to stimulate oil accumulation without a growth penalty or diminished biomass accumulation has been a major goal of the research community. Indeed, a major driver of the US Department of Energy Aquatic Species Program (Sheehan, 1998) was the identification of a hypothetical “lipid trigger” that could be induced under optimal growth conditions, leading to the reproducible and high-yielding production of desired products. This hypothetical trigger has proven elusive, and the manipulation of single enzymes or transcriptional regulators has been only marginally effective in achieving the desired goals (Ajjawi et al., 2017).

Another recently proposed approach for the “lipid trigger” is the use of synthetic chemicals that induce storage lipid production with minimal retardation in growth, although the precise molecular mechanisms by which these compounds induce lipogenesis are, at present, poorly defined (Wase et al., 2014; Wase et al., 2015; Wase et al., 2018; Wase et al., 2019). Nevertheless, significant progress has been made in understanding the enzymology and certain aspects of the regulation of TAG biosynthesis and degradation in algal model systems such as Chlamydomonas (Li-Beisson et al., 2015; Li-Beisson et al., 2019) and *Nannochloropsis* (Ma X. N. et al., 2016). Clearly, the existence and nature of a hypothetical “lipid trigger” remain tenuous. This study presents several new insights related to abiotic stress signals that likely play an underappreciated role in stress-induced TAG accumulation in oleaginous algae.

**Figure 7:**
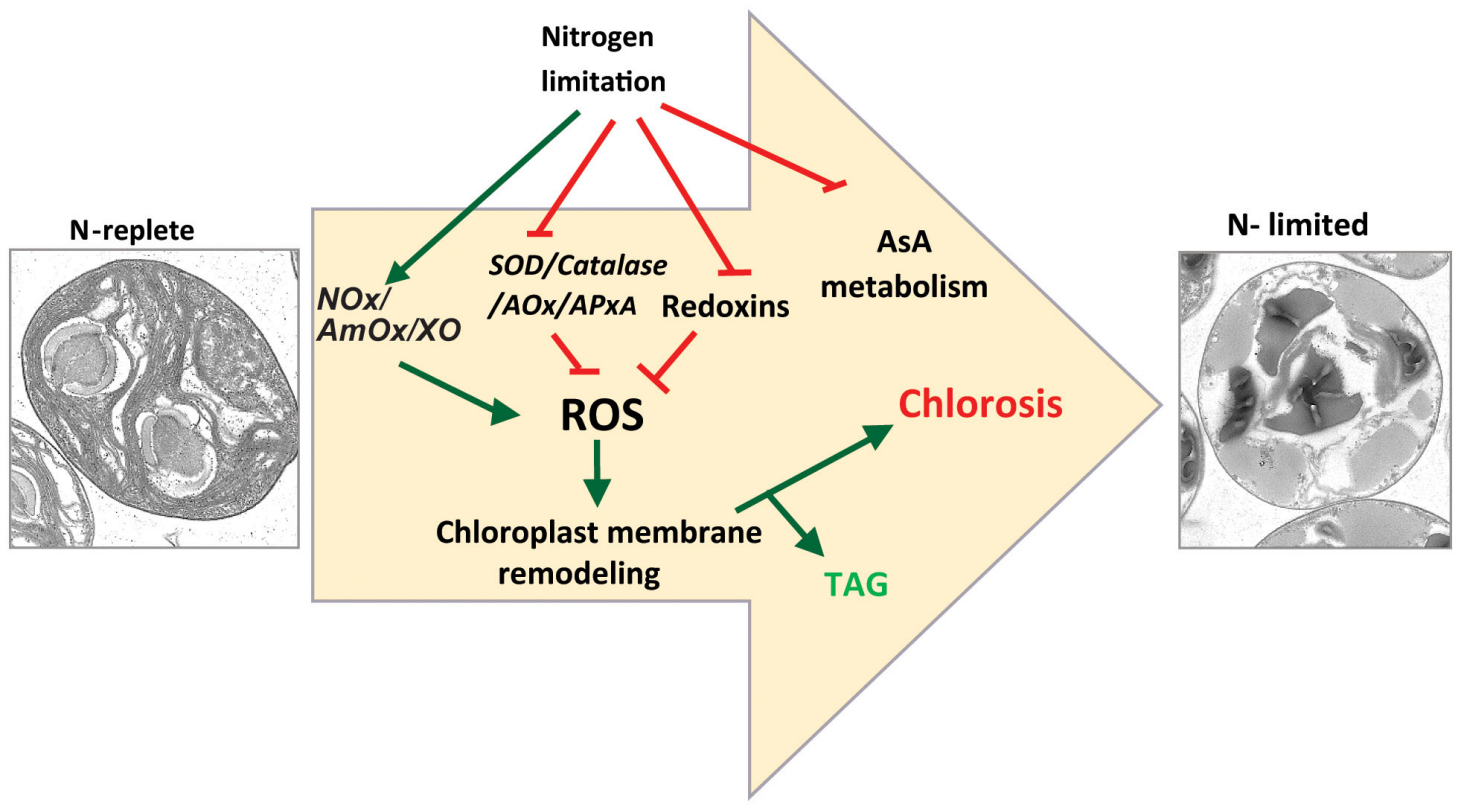
Proposed model for nitrogen starvation mediated ROS accumulation leading to chlorosis and TAG accumulation. NOx, NADPH oxidase; AmOx, amine oxidase; XO, xanthine oxidase; SOD, superoxide dismutase; AOx, alternate oxidase; APx, ascorbate oxidase.

First, we showed that, as in other algal species, N-limitation in *C. sorokiniana* UTEX-1230 is a potent inducer of thylakoid membrane degradation and channeling of fatty acids from chloroplast lipids into TAG (**Figure 7**). Our transcriptome analysis revealed that the expression of genes encoding many key enzymes involved in regulating ROS levels in cells, such as superoxide dismutase, catalase, and stromal ascorbate peroxidase, is downregulated, concomitantly with an increase in ROS levels under N limitation. This observation strongly suggests that cells actively accumulate ROS in a regulated fashion under these conditions. This is achieved by lowering the transcript abundance of enzymes involved in ROS quenching. The DCFDA assay provides a quantitative measurement of total ROS but does not indicate which specific ROS species (superoxides, hydrogen peroxide, or others) accumulate or which is linked to membrane remodeling and TAG accumulation. It is also possible that each ROS species plays an independent role in the various branches of the N starvation response.

We cannot ascertain the subcellular localization of ROS quenching enzymes, as *C. sorokiniana* is not yet amenable to genetic transformation. However, the separation of cells into various organelle preparation and biochemical assays showed that a Fe-SOD is present in the chloroplast, whereas a Mn-SOD is present in mitochondria in Chlamydomonas (Kitayama et al., 1999). In the case of *C. sorokiniana*, we determined that the expression of the genes encoding both Fe-SOD (*SOD2* and *SOD3*) and Mn-SOD (*SOD1*) is downregulated upon N limitation. While we observed that the expression of *SOD4*, encoding a Mn-SOD, increased under our N-limited conditions, the relevance of this increase is unclear. Catalase is a mitochondrial protein in Chlamydomonas (Kato et al., 1997). The lower expression of genes encoding ROS-scavenging enzymes localizing to both chloroplast and mitochondria indicates the possibility that more than one organelle may be a source of ROS under N-limiting conditions.

The expression of genes encoding multiple ferredoxins and peroxiredoxins known to be involved in maintaining redox homeostasis changed upon N limitation. Additionally, we observed that the expression of genes related to ROS generation such as *Amine oxidase*s, *Xanthine oxidase*, and *NOX*s increased. This result indicated that *C. sorokiniana* cells may undertake a two-pronged approach to ROS accumulation by downregulating scavenging systems and upregulating ROS-producing processes, further indicating that ROS accumulation is an active and regulated process.

The data presented in this study pertaining to ROS accumulation and redox signaling are based on gene expression profiles, which may not always correlate with the abundance or the activity of the gene product, which can depend on absolute protein levels and protein modifications. However, we saw a coordinated decrease in the expression levels of many key genes related to ROS accumulation and redox signaling. These observations, coupled with an increase in ROS levels in the cell, indicate that this response is likely a transcriptionally regulated process and that there is an underappreciated role for ROS and redox signaling under N limitation. Higher ROS levels under N limitation were also observed in the diatom *Phaeodactylum tricornutum* (Mizrachi et al., 2019). Accumulation of ROS under N limitation is thus not limited to *C. sorokiniana* and may confer a physiological advantage that has enabled this phenotype to be selected and maintained in these distantly related photosynthetic eukaryotes.

The ectopic induction of ROS accumulation by inhibiting SOD leads to MGDG degradation, whereas ROS quenching under N limitation limits MGDG degradation. The concomitant decrease in TAG levels in ascorbate-treated N-limited cells is thus likely due to a lower rate of degradation of membrane lipids such as MGDG and phospholipids. From these observations, it is clear that membrane remodeling–mediated TAG accumulation is closely linked to redox signaling. This result is in line with Zhang et al. (2013) who observed that exogenous application of hydrogen peroxide was sufficient to induce TAG accumulation in *C. sorokiniana C3.* In Chlamydomonas, a substantial flux of fatty acid for TAG accumulation is derived from preexisting glycerolipids (Young and Shachar-Hill, 2021). Indeed, mutations in genes encoding enzymes such as PGD1 or PDAT1 lead to lower TAG accumulation under N limitation. PGD1 and PDAT1 enzymes are involved in the turnover of fatty acid from MGDG and phosphatidylcholine to TAG, respectively (Li et al., 2012b; Yoon et al., 2012). In sum, our study strongly implicates N-limitation induced ROS accumulation as playing a crucial role in the metabolic reprogramming that leads to TAG accumulation (**Figure 7**). Future studies will be aimed at identifying the components that sense N limitation and alter ROS accumulation, as well as identifying the enzymes that act downstream of the ROS burst to regulate membrane turnover and TAG accumulation, thus providing additional details regarding the molecular trigger(s) for macronutrient-limited TAG accumulation in microalgae.

## Data availability statement

The data presented in the study are deposited in the NCBI repository, accession number PRJNA1111962.

## Author contributions

JV: Conceptualization, Data curation, Investigation, Methodology, Visualization, Writing – original draft, Writing – review & editing. NW: Data curation, Methodology, Visualization, Writing – review & editing. KL: Methodology, Resources, Writing – review & editing. WM: Methodology, Writing – review & editing. CZ: Methodology, Resources, Writing – review & editing. WR: Conceptualization, Funding acquisition, Investigation, Supervision, Writing – review & editing.

## References

[B1] Ajjawi I. Verruto J. Aqui M. Soriaga L. B. Coppersmith J. Kwok K.. (2017). Lipid production in *Nannochloropsis gaditana* is doubled by decreasing expression of a single transcriptional regulator. Nat. Biotechnol. 35, 647–652. doi: 10.1038/nbt.3865 28628130

[B2] Allen J. Black P. N. Riekhof W. R. Allen J. Unlu S. Demirel Y.. (2018). Integration of biology, ecology and engineering for sustainable algal-based biofuel and bioproduct biorefinery Integration of biology, ecology and engineering for sustainable algal-based biofuel and bioproduct biorefinery. Bioresour. Bioprocess. 5, 47 doi: 10.1186/s40643-018-0233-5

[B3] Altenhöfer S. Radermacher K. A. Kleikers P. W. M. Wingler K. Schmidt H. H. H. W. (2015). Evolution of NADPH oxidase inhibitors: Selectivity and mechanisms for target engagement. Antioxidants Redox Signaling 23, 406–427. doi: 10.1089/ars.2013.5814 24383718 PMC4543484

[B4] Asada K. (2006). Production and scavenging of reactive oxygen species in chloroplasts and their functions. Plant Physiol. 141, 391–396. doi: 10.1104/pp.106.082040 16760493 PMC1475469

[B5] Baumann U. Juttner J. (2002). Plant thioredoxins: the multiplicity conundrum. CMLS, Cell. Mol. Life Sci. 59, 1042–1057. doi: 10.1007/s00018-002-8485-8 12169016 PMC11337411

[B6] Ben Rejeb K. Benzarti M. Debez A. Bailly C. Savouré A. Abdelly C. (2015). NADPH oxidase-dependent H_2_O_2_ production is required for salt-induced antioxidant defense in Arabidopsis thaliana. J. Plant Physiol. 174, 5–15. doi: 10.1016/j.jplph.2014.08.022 25462961

[B7] Boyle N. R. Page M. D. Liu B. Blaby I. K. Casero D. Kropat J.. (2012). Three acyltransferases and nitrogen-responsive regulator are implicated in nitrogen starvation-induced triacylglycerol accumulation in Chlamydomonas. J. Biol. Chem. 287, 15811–15825. doi: 10.1074/jbc.M111.334052 22403401 PMC3346115

[B8] Czarnocka W. Karpiński S. (2018). Friend or foe? Reactive oxygen species production, scavenging and signaling in plant response to environmental stresses. Free Radical Biol. Med. 122, 4–20. doi: 10.1016/j.freeradbiomed.2018.01.011 29331649

[B9] Foyer C. H. Noctor G. (2009). Redox regulation in photosynthetic organisms: Signaling, Acclimation, and Practical Implications. Antioxidants & Redox Signaling 11, 861–905. doi: 10.1089/ars.2008.2177 19239350

[B10] Gianni D. Taulet N. Zhang H. Dermardirossian C. Kister J. Martinez L. (2010). A novel and specific NADPH oxidase-1 (Nox1) small-molecule inhibitor blocks the formation of functional invadopodia in human colon cancer cells. ACS Chem. Biol. 5, 981–993. doi: 10.1021/cb100219n 20715845 PMC2955773

[B11] Gojkovic Ž. Vílchez C. Torronteras R. Vigara J. Gómez-Jacinto V. Janzer N.. (2014). Effect of selenate on viability and selenomethionine accumulation of Chlorella sorokiniana grown in batch culture. Sci. World J. 401265, 13. doi: 10.1155/2014/401265 PMC3928859 24688385

[B12] Gorman D. S. Levine R. P. (1965). Cytochrome f and plastocyanin: their sequence in the photosynthetic electron transport chain of Chlamydomonas reinhardii. Proc. Natl. Acad. Sci. U.S.A 54, 1665–1669. doi: 10.1073/pnas.54.6.1665 4379719 PMC300531

[B13] Gupta D. K. Palma J. M. Corpas F. J. (2013). Heavy Metal Stress In Plants Springer. doi: 10.1007/978-3-642-38469-1

[B14] He H. Yan J. Yu X. Liang Y. Fang L. Scheller H. V.. (2017). The NADPH-oxidase AtRbohI plays a positive role in drought-stress response in Arabidopsis thaliana. Biochem. Biophys. Res. Commun. 491, 834–839. doi: 10.1016/j.bbrc.2017.05.131 28559135

[B15] Heikkila R. E. Cabbat F. S. Cohen G. (1976). *In vivo* inhibition of superoxide dismutase in mice by diethyldithiocarbamate. J. Biol. Chem. 251, 2182–2185. doi: 10.1016/S0021-9258(17)33675-X 5443

[B16] Hu Q. Sommerfeld M. Jarvis E. Ghirardi M. Posewitz M. Seibert M.. (2008). Microalgal triacylglycerols as feedstocks for biofuel production: Perspectives and advances. Plant J. 54, 621–639. doi: 10.1111/j.1365-313X.2008.03492.x 18476868

[B17] Jiang G. Li F. Wang S. Sun J. Liu Z. (2019). Positive correlation between lipid accumulation and gene expression of a copper-containing amine oxidase gene in Chlorella under nitrogen starvation. Algal Res. 40, 101504. doi: 10.1016/j.algal.2019.101504

[B18] Kato J. Yamahara T. Tanaka K. Takio S. Satoh T. (1997). Characterization of catalase from green algae Chlamydomonas reinhardtii. J. Plant Physiol. 151, 262–268. doi: 10.1016/S0176-1617(97)80251-9

[B19] Kitayama K. Kitayama M. Togasaki R. K. (1999). Subcellular localization of iron and manganese superoxide dismutase in *Chlamydomonas reinhardtii* (chlorophyceae). J. Phycology. 142, 136–142. doi: 10.1046/j.1529-8817.1999.3510136.x

[B20] Li X. Benning C. Kuo M. H. (2012a). Rapid triacylglycerol turnover in *Chlamydomonas reinhardtii* requires a lipase with broad substrate specificity. Eukaryotic Cell. 11, 1451–1462. doi: 10.1128/EC.00268-12 23042128 PMC3536278

[B21] Li X. Moellering E. R. Liu B. Johnny C. Fedewa M. Sears B. B.. (2012b). A galactoglycerolipid lipase is required for triacylglycerol accumulation and survival following nitrogen deprivation in Chlamydomonas reinhardtii. Plant Cell 24, 4670–4686. doi: 10.1105/tpc.17.00446 23161887 PMC3531859

[B22] Liao Y. Smyth G. K. Shi W. (2013). The Subread aligner: Fast, accurate and scalable read mapping by seed-and-vote. Nucleic Acids Res. 41 (10), e108. doi: 10.1093/nar/gkt214 23558742 PMC3664803

[B23] Li-Beisson Y. Beisson F. Riekhof W. (2015). Metabolism of acyl-lipids in Chlamydomonas reinhardtii. Plant J. 82, 504–522. doi: 10.1111/tpj.12787 25660108

[B24] Li-Beisson Y. Thelen J. J. Fedosejevs E. Harwood J. L. (2019). The lipid biochemistry of eukaryotic algae. Prog. Lipid Res. 74, 31–68. doi: 10.1016/j.plipres.2019.01.003 30703388

[B25] Ma X. N. Chen T. P. Yang B. Liu J. Chen F. (2016). Lipid production from nannochloropsis. Mar. Drugs 14. doi: 10.3390/md14040061 PMC4849066 27023568

[B26] Ma X. Wang W. Bittner F. Schmidt N. Berkey R. Zhang L.. (2016). Dual and opposing roles of xanthine dehydrogenase in defense-associated reactive oxygen species metabolism in arabidopsis. Plant Cell. 28, 1108–1126. doi: 10.1105/tpc.15.00880 27152019 PMC4904670

[B27] Meitzler J. L. Antony S. Wu Y. Juhasz A. Liu H. Jiang G.. (2014). NADPH oxidases: A perspective on reactive oxygen species production in tumor biology. Antioxidants Redox Signaling 20, 2873–2889. doi: 10.1089/ars.2013.5603 24156355 PMC4026372

[B28] Meyer Y. Siala W. Bashandy T. Riondet C. Vignols F. Reichheld J. P. (2008). Glutaredoxins and thioredoxins in plants. Biochim. Biophys. Acta - Mol. Cell Res. 1783, 589–600. doi: 10.1016/j.bbamcr.2007.10.017 18047840

[B29] Miller R. Wu G. Deshpande R. R. Vieler A. Gartner K. Li X.. (2010). Changes in transcript abundance in *Chlamydomonas reinhardtii* following nitrogen deprivation predict diversion of metabolism. Plant Physiol. 154, 1737–1752. doi: 10.1104/pp.110.165159 20935180 PMC2996024

[B30] Mizrachi A. Graff van Creveld S. Shapiro O. H. Rosenwasser S. Vardi A. (2019). Light-dependent single-cell heterogeneity in the chloroplast redox state regulates cell fate in a marine diatom. eLife. 8, 1–27. doi: 10.7554/eLife.47732 PMC6682412 31232691

[B31] Møller I. M. (2001). P LANT M ITOCHONDRIA AND O XIDATIVE S TRESS: electron transport, NADPH turnover, and metabolism of reactive oxygen species. Annu. Rev. Plant Physiol. Plant Mol. Biol. 52, 561–591. doi: 10.1146/annurev.arplant.52.1.561 11337409

[B32] Rhee S. G. Woo H. A. (2011). Multiple functions of peroxiredoxins: Peroxidases, sensors and regulators of the intracellular messenger H2O2, and protein chaperones. Antioxidants Redox Signaling 15, 781–794. doi: 10.1089/ars.2010.3393 20919930

[B33] Ritchie M. E. Phipson B. Wu D. Hu Y. Law C. W. Shi W.. (2015). Limma powers differential expression analyses for RNA-sequencing and microarray studies. Nucleic Acids Res. 43, e47. doi: 10.1093/nar/gkv007 25605792 PMC4402510

[B34] Robinson M. D. McCarthy D. J. Smyth G. K. (2009). edgeR: A Bioconductor package for differential expression analysis of digital gene expression data. Bioinformatics. 26, 139–140. doi: 10.1093/bioinformatics/btp616 19910308 PMC2796818

[B35] Schieber M. Chandel N. S. (2014). ROS function in redox signaling and oxidative stress. CURBIO. 24, R453–R462. doi: 10.1016/j.cub.2014.03.034 PMC4055301 24845678

[B36] Schürmann P. Buchanan B. B. (2008). The ferredoxin/thioredoxin system of oxygenic photosynthesis. Antioxidants Redox Signaling 10, 1235–1273. doi: 10.1089/ars.2007.1931 18377232

[B37] Sheehan J. Dunahay T. Benemann J. Roessler P. (1998). A look back at the u. s. department of energy’s aquatic species program: biodiesel from algae. Natl. Renewable Energy Lab.

[B38] Shtaida N. Khozin-Goldberg I. Solovchenko A. Chekanov K. Didi-Cohen S. Leu S.. (2014). Downregulation of a putative plastid PDC E1α subunit impairs photosynthetic activity and triacylglycerol accumulation in nitrogen-starved photoautotrophic Chlamydomonas reinhardtii. J. Exp. Bot. 65, 6563–6576. doi: 10.1093/jxb/eru374 25210079 PMC4246187

[B39] Smirnoff N. (2018). Ascorbic acid metabolism and functions: A comparison of plants and mammals. Free Radical Biol. Med. 122, 116–129. doi: 10.1016/j.freeradbiomed.2018.03.033 29567393 PMC6191929

[B40] Stephenson A. L. Dennis J. S. Howe C. J. Scott S. A. Smith A. G. (2010). Influence of nitrogen-limitation regime on the production by Chlorella vulgaris of lipids for biodiesel feedstocks. Biofuels. 1, 47–58. doi: 10.4155/bfs.09.1

[B41] Tavladoraki P. Cona A. Angelini R. (2016). Copper-containing amine oxidases and FAD-dependent polyamine oxidases are key players in plant tissue differentiation and organ development. Front. Plant Sci. 7. doi: 10.3389/fpls.2016.00824 PMC4923165 27446096

[B42] Vijayan J. Wase N. Liu K. Zhang C. Riekhof W. R. (2021). Reactive oxygen species mediate thylakoid membrane remodeling and triacylglycerol synthesis under nitrogen starvation in the alga *Chlorella sorokiniana* . bioRxiv Preprint. doi: 10.1101/2021.05.15.444036 PMC11261311 39040507

[B43] Wase N. Black P. DiRusso C. (2018). Innovations in improving lipid production: Algal chemical genetics. Prog. Lipid Res. 71, 101–123. doi: 10.1016/j.plipres.2018.07.001 30017715

[B44] Wase N. Black P. N. Stanley B. A. Dirusso C. C. (2014). Integrated quantitative analysis of nitrogen stress response in *Chlamydomonas reinhardtii* using metabolite and protein profiling. J. Proteome Res. 13, 1373–1396. doi: 10.1021/pr400952z 24528286

[B45] Wase N. Tu B. Black P. N. DiRusso C. C. (2015). Phenotypic screening identifies Brefeldin A/Ascotoxin as an inducer of lipid storage in the algae Chlamydomonas reinhardtii. Algal Res. 11, 74–84. doi: 10.1016/j.algal.2015.06.002

[B46] Wase N. Tu B. Allen J. W. Black P. N. DiRusso C. C. (2017). Identification and metabolite profiling of chemical activators of lipid accumulation in green algae. Plant Physiol. 174, 2146–2165. doi: 10.1104/pp.17.00433 28652262 PMC5543952

[B47] Wase N. Tu B. Rasineni G. K. Cerny R. Grove R. Adamec J.. (2019). Remodeling of Chlamydomonas metabolism using synthetic inducers results in lipid storage during growth. Plant Physiol. 181, 00758.2019. doi: 10.1104/pp.19.00758 PMC6836844 31501300

[B48] Yoon K. Han D. Li Y. Sommerfeld M. Hu Q. (2012). Phospholipid: Diacylglycerol acyltransferase is a multifunctional enzyme involved in membrane lipid turnover and degradation while synthesizing triacylglycerol in the unicellular green microalga Chlamydomonas reinhardtii. Plant Cell 24, 3708–3724. doi: 10.1105/tpc.112.100701 23012436 PMC3480297

[B49] Young D. Y. Shachar-Hill Y. (2021). Large fluxes of fatty acids from membranes to triacylglycerol and back during N-deprivation and recovery in Chlamydomonas. Plant Physiol. 185, 796–814. doi: 10.1093/plphys/kiaa071 33822218 PMC8133548

[B50] Zandalinas S. I. Mittler R. (2018). ROS-induced ROS release in plant and animal cells. Free Radical Biol. Med. 122, 21–27. doi: 10.1016/j.freeradbiomed.2017.11.028 29203327

[B51] Zhang Y. M. Chen H. He C. L. Wang Q. (2013). Nitrogen starvation induced oxidative stress in an oil-producing green alga *chlorella sorokiniana* C3. PloS One 8, 1–12. doi: 10.1371/journal.pone.0069225 PMC3712941 23874918

[B52] Zhu L. D. Li Z. H. Hiltunen E. (2016). Strategies for lipid production improvement in microalgae as a biodiesel feedstock. BioMed. Res. Int. 2016, 7–9. doi: 10.1155/2016/8792548 PMC5048031 27725942

