# Supplementary figures and images for: ROS-mediated thylakoid membrane remodeling and triacylglycerol biosynthesis under nitrogen starvation in the alga Chlorella sorokiniana
Source: Front Plant Sci. 2024 Jul 8;15:1418049. doi: 10.3389/fpls.2024.1418049 (PMC11261311; doi:10.3389/fpls.2024.1418049)

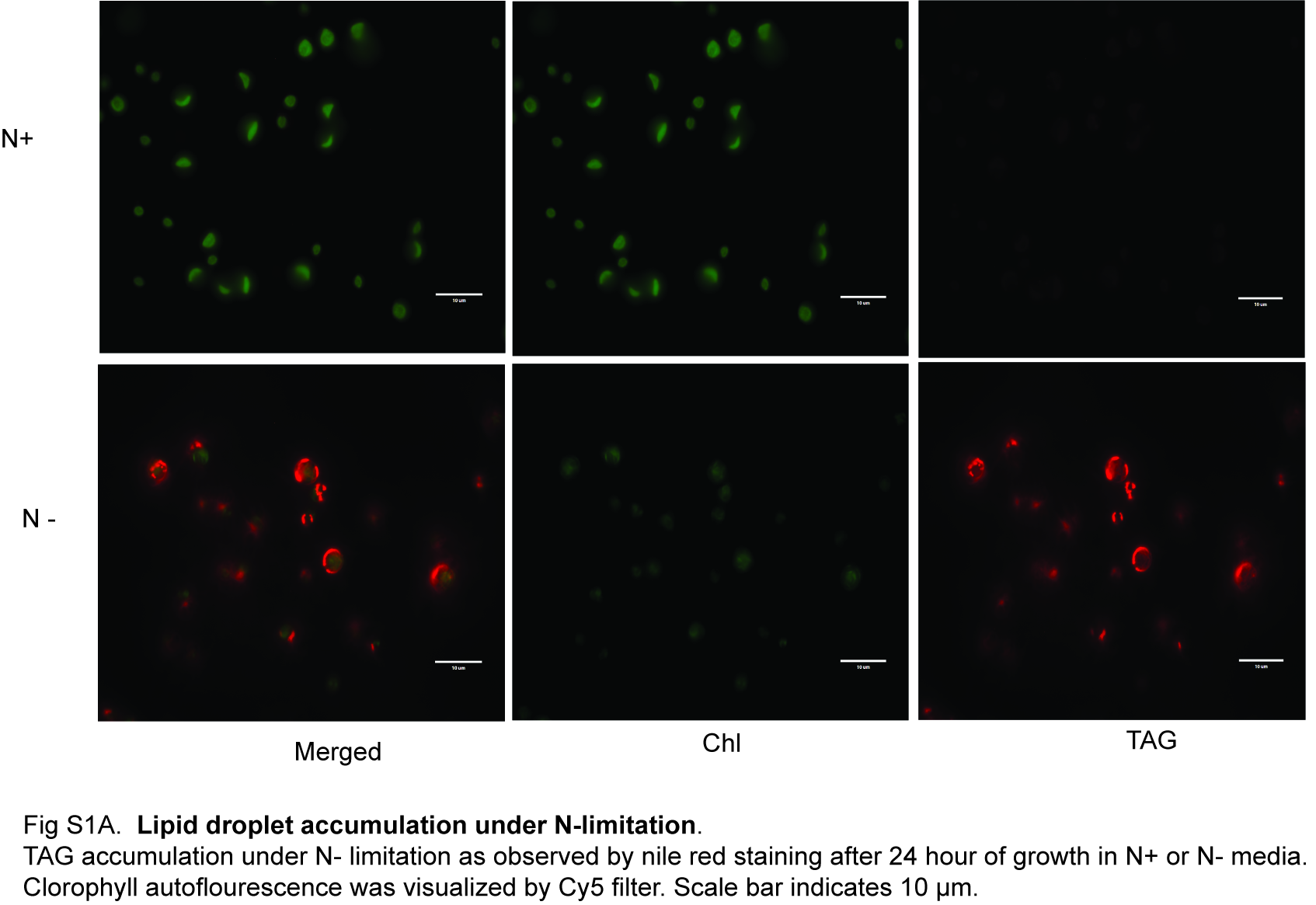

Supplement: Supplementary file 2 [file Image_1.tif]

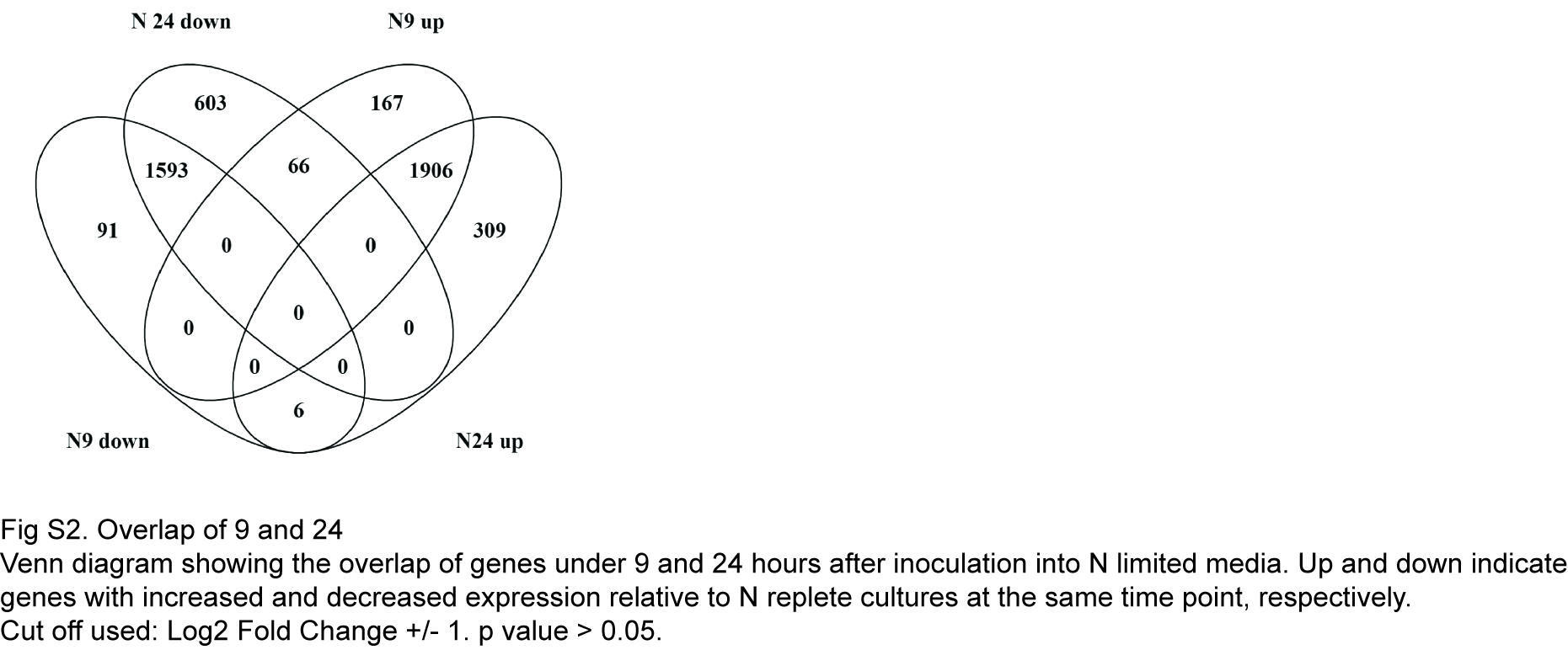

Supplement: Supplementary file 3 [file Image_2.tif]

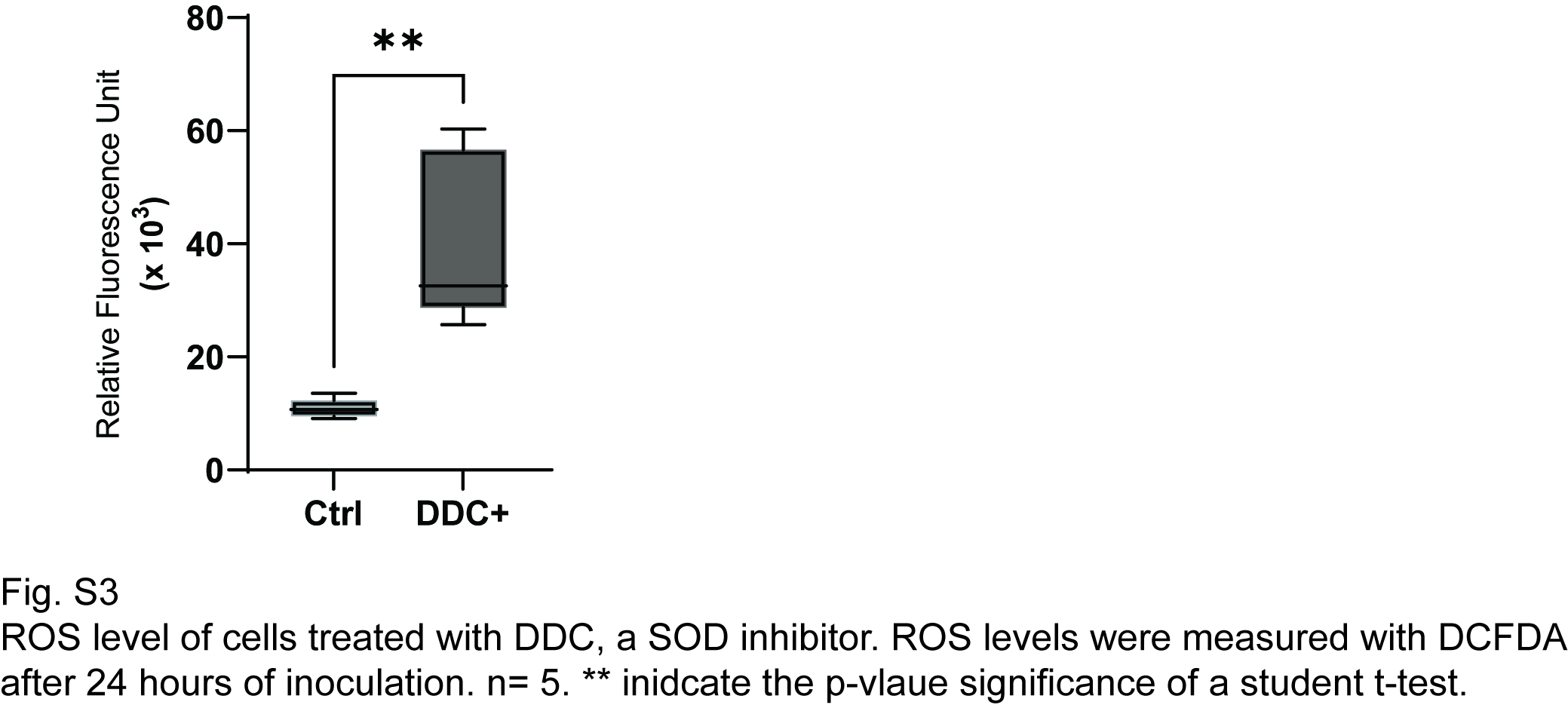

Supplement: Supplementary file 4 [file Image_3.tif]
